# Generalized herpes zoster infection with left vocal fold paralysis in a patient with immunosuppression

**DOI:** 10.1186/1471-2334-13-S1-P51

**Published:** 2013-12-16

**Authors:** Michaela Oana, Doina Baltaru, Yveta Iernuțan, Felician Chirteş, Simona Costin, Mihai Feier

**Affiliations:** 1 Emergency Military Hospital “C-tin Papilian”, Cluj-Napoca, Romania

## Case report

We present the case of a 62 year-old man known with rheumatoid arthritis (treated with methotrexate), mechanic mitral valve prosthesis (for mitral insufficiency secondary to infectious endocarditis), Hodgkin lymphoma in complete remission, repetitive ischemic strokes, who was admitted on 30.06.2013 to the Department of Infectious Diseases of our hospital for the appearance of a generalized papulo-vesicular rash, buccal aphta, dysphagia, loss of appetite. At the time of admission, laboratory findings showed mild leukocytosis, inflammatory syndrome, ketoacidosis, a prolonged INR in the context of over dosage of coumarins.

Based on the clinical exam and on the histopathology from the base of a vesicle the diagnosis of generalized herpes zoster infection was established. The infection was favored by the immunosuppression secondary to his diseases but also by the treatment with methotrexate.

During his hospital admission he developed dysphagia especially for liquids, dysphonia. The otolaryngology examination revealed the paralysis of the left vocal fold. The CT scan of the thorax and abdomen did not find lesions suggestive for recurrence of the lymphoma.

The patient was treated with acyclovir (i.v. first and p.o after a few days) for a period of 10 days, augmentin for 7 days. He received also parenteral hydration, nootropics, anticoagulants, proton-pump inhibitors, aerosols with ventolin, spiriva and respiratory kinetotherapy. The evolution of the skin rash was favorable but with the persistence of the paralysis of the left vocal fold evidenced by laryngoscopy.

